# Abstract

**Published:** 1914-05

**Authors:** 


Robin (*Traitment de la Tuberculose*) holds that an adequate supply of mineral substances is an indispensable factor in the successful treatment of tuberculosis, and suggests the following formula:

| | |
|---|---|
| Bone, freshly powdered............ | 1.0 gm. |
| Precipitated chalk ................ | 0.4 gm. |
| Magnesium carbonate ............. | 0.1 gm. |
| Milk sugar ........................ | 1.0 gm. |
| Sodium bicarbonate ............... | 0.5 gm. |

This quantity may be given twice daily after meals.

